# Harnessing citizen science in health promotion: perspectives of policy and practice stakeholders in Australia

**DOI:** 10.1093/heapro/daad101

**Published:** 2023-09-14

**Authors:** Yvonne Laird, Leah Marks, Ben J Smith, Pippy Walker, Kate Garvey, Kim Jose, Sean O’Rourke, Katherine Pontifex, Karen Wardle, Samantha Rowbotham

**Affiliations:** Prevention Research Collaboration, School of Public Health, Faculty of Medicine and Health, The University of Sydney, Sydney, New South Wales, Australia; Charles Perkins Centre, The University of Sydney, Sydney, New South Wales, Australia; The Australian Prevention Partnership Centre, The Sax Institute, Sydney, New South Wales, Australia; Charles Perkins Centre, The University of Sydney, Sydney, New South Wales, Australia; The Australian Prevention Partnership Centre, The Sax Institute, Sydney, New South Wales, Australia; Menzies Centre for Health Policy and Economics, School of Public Health, Faculty of Medicine and Health, The University of Sydney, Sydney, New South Wales, Australia; Prevention Research Collaboration, School of Public Health, Faculty of Medicine and Health, The University of Sydney, Sydney, New South Wales, Australia; Charles Perkins Centre, The University of Sydney, Sydney, New South Wales, Australia; The Australian Prevention Partnership Centre, The Sax Institute, Sydney, New South Wales, Australia; Charles Perkins Centre, The University of Sydney, Sydney, New South Wales, Australia; The Australian Prevention Partnership Centre, The Sax Institute, Sydney, New South Wales, Australia; Menzies Centre for Health Policy and Economics, School of Public Health, Faculty of Medicine and Health, The University of Sydney, Sydney, New South Wales, Australia; Department of Health, Tasmanian Government, Hobart, Tasmania, Australia; School of Psychology and Public Health, La Trobe University, Melbourne, Victoria, Australia; Menzies Institute for Medical Research, University of Tasmania, Hobart, Tasmania, Australia; VicHealth, West Melbourne, Victoria, Australia; Wellbeing SA, Adelaide, South Australia, Australia; South Western Sydney Local Health District, Warwick Farm, New South Wales, Australia; Charles Perkins Centre, The University of Sydney, Sydney, New South Wales, Australia; The Australian Prevention Partnership Centre, The Sax Institute, Sydney, New South Wales, Australia; Menzies Centre for Health Policy and Economics, School of Public Health, Faculty of Medicine and Health, The University of Sydney, Sydney, New South Wales, Australia

**Keywords:** citizen science, public health, capacity building, community engagement, health behaviour

## Abstract

Citizen science is rapidly gaining momentum as a means of involving members of the public in research and decision-making in disease prevention and health promotion. However, citizen science projects have predominantly been led by academic researchers and there is limited understanding of how to support the application of citizen science approaches in policy and practice settings. This study aimed to understand the perceptions, motivations and early experiences of applying citizen science approaches in policy and practice settings. Semi-structured interviews were conducted with policy and practice stakeholders who were leading citizen science projects (project partners, *n* = 7), and their implementation partners (project implementers, *n* = 11). Participants viewed citizen science as an opportunity to access hard-to-reach data and to enhance engagement with community members to support policy and practice change. Barriers and facilitators of citizen science in policy and practice settings included navigating collaborative relationships, team capacity and resources available to deliver projects, recruitment and engagement of citizen scientists and ethical considerations in the design and implementation of citizen science projects. Findings support the feasibility and wider application of citizen science approaches in health promotion and are being used to inform the development of tools and resources to build capacity in these approaches in policy and practice settings.

**Contribution to Health Promotion**

- Citizen science has potential to improve the relevance of research and decision-making in health promotion.
- Our findings highlight considerations for the expanded use of citizen science in policy and practice settings in health promotion.
- Successful implementation of citizen science requires appropriate team capacity, skills and resources and effective collaborative relationships.
- Design of citizen science projects should identify strategies to recruit and retain citizen scientists and address potential ethical concerns such as safety.
- Findings have informed the development of tools and resources to build capacity in citizen science in policy and practice settings.

## INTRODUCTION

Preventable morbidity and mortality, largely due to chronic diseases, are responsible for substantial social and economic costs in Australia (Crosland *et al.*, 2019; Australian Institute of Health and Welfare, 2020). The drivers of this burden of disease are complex and multifaceted, requiring multi-sectoral and co-ordinated action at multiple levels to develop sustainable and acceptable solutions (Frieden, 2014; Wutzke *et al.*, 2017). Public engagement is recognized as a vital strategy to strengthen research-informed policy and practice in health promotion in Australia (National Health and Medical Research Council, 2016) and globally (World Health Organization, 2017), building on a long history of community engagement in public health practice. There are a myriad of approaches to engaging the public in health promotion, such as co-production and community consultations, although there is limited information to guide policy and practice stakeholders in determining which approach to use, for what purpose and the relative benefits and challenges of different approaches (Pytlikzillig and Tomkins, 2011; Domecq *et al.*, 2014; Marks *et al.*, 2023).

Citizen science, defined as active involvement of members of the public (citizen scientists) in the research process (Shanley *et al.*, 2019), has origins in the natural sciences but is growing in popularity in health and other disciplines for its potential to enhance the relevance of research and decision-making (Follett and Strezov, 2015; Marks *et al.*, 2022). Citizen scientists can be involved in multiple phases of the research process, from identifying priorities for research, contributing to research design, collecting and/or analysing data and contributing to the publication and dissemination of findings, although an active involvement in the collection and analysis of data is often a key component of citizen science (Bonney *et al.*, 2009; Kullenberg and Kasperowski, 2016). Through harnessing the expertise, skills and passions of members of the public, citizen science approaches have the potential to provide access to volumes and types of data that may otherwise be difficult to obtain. These data can inform the development of solutions that meet the needs of community members (De Sherbinin *et al.*, 2021). Involving members of the public in research also has the potential to build skills and scientific literacy of community members and generate increased awareness, support and advocacy for action to address issues of concern to them (Bonney *et al.*, 2016; Den Broeder *et al.*, 2017).

However, citizen science initiatives in disease prevention and health promotion have predominantly been led by academic researchers (Marks *et al.*, 2022) and there is limited understanding about how these approaches are perceived by policy and practice stakeholders and how to support their application in policy and practice settings. This study aimed to address gaps in the literature to understand how policy and practice stakeholders in health promotion perceive citizen science, their motivations for utilizing citizen science, and factors supporting or inhibiting implementation of citizen science approaches. Information generated from this research will provide insights on the feasibility and suitability of applying citizen science approaches in policy and practice settings in health promotion and guide the development of tools and resources to support the broader use of citizen science in these settings.

## METHOD

This qualitative study is part of a broader program of work, the ‘Citizen Science in Prevention Project’, described below. Individual interviews were conducted at the early stages of implementation of four citizen science projects as part of a developmental evaluation (Patton, 2010; Van Stolk and Ling, 2020) to share insights within and across the four projects and inform the provision of support and capacity building activities. Findings are reported in line with the Consolidated Criteria for Reporting Qualitative Research (COREQ) guidelines (Tong *et al.*, 2007). Ethical approval for this study was granted by the University of Sydney Human Research Ethics Committee (REF: 2020/647).

### The Citizen Science in Prevention Project

The Citizen Science in Prevention Project is a co-produced program of work funded by the Australian Prevention Partnership Centre that was established to build capacity in the application of citizen science approaches in policy and practice in disease prevention and health promotion. Amongst other activities, this includes supporting the development and implementation of four citizen science projects being led by health promotion agencies across Australia [South Western Sydney Local Health District, Tasmanian Department of Health, Public Health Services, the Victorian Health Promotion Foundation (VicHealth) and Wellbeing SA]. By working closely with these stakeholders (our ‘project partners’), the project aimed to inform the broader application of citizen science approaches in policy and practice settings. Full details of the program of work and evaluation are reported elsewhere (Rowbotham *et al.*, 2022). Briefly, following discussions with the research team, each project partner identified and resourced their own citizen science project in disease prevention and health promotion as follows:

1) South Western Sydney Local Health District aimed to use citizen science to evaluate health-related impacts of an urban regeneration initiative;
2) Tasmanian Department of Health, Public Health Services, applied a citizen science approach to monitor the walkability of rural towns in Tasmania;
3) VicHealth used citizen science to monitor harmful industry marketing that young people were being exposed to online; and
4) Wellbeing SA applied a citizen science approach to evaluate the accessibility of two community garden initiatives.

### Recruitment

All team members from each of the four citizen science projects (our ‘cases’) as part of the Citizen Science in Prevention Project were invited to take part. This included policy and practice stakeholders who were leading each of the four citizen science projects (project partner, *n* = 8) and university and local government stakeholders who were responsible for implementing the projects (project implementer, *n* = 13).

### Interview guide

A semi-structured interview guide was developed to gain insights around stakeholders’ familiarity with and perceptions of citizen science, why they chose to apply a citizen science approach in their projects, and reflections on factors that supported or inhibited the implementation of each citizen science project. Interviews were conducted online via videoconferencing by two team members (Y.L. and L.M.) between December 2020 and September 2021. At the time of interviews, projects were at different stages of implementation with one project preparing to commence recruitment, one actively recruiting, one in data analysis and one at the point of disseminating project reports to their communities and funding partners. In line with developmental evaluation, the researchers conducting interviews were embedded evaluators rather than objective outsiders, meaning they had familiarity with each project and an existing relationship with some participants (e.g. project partners) prior to interviews. Interviews were an average of 44 minutes in duration (range 31–62 min). Participant names are replaced with pseudonyms throughout, corresponding to their role on the project (project partner = PP, project implementer = PI).

### Analysis

Interview transcripts were imported into NVivo qualitative data analysis software (Qsr International Pty Ltd, 2018) and analysed inductively using thematic analysis (Braun and Clarke, 2021). Data analysis was led by two members of the research team (Y.L. and L.M.). Five team members (Y.L., L.M., S.R., B.S. and P.W.) independently coded four transcripts and met to discuss initial codes. These initial codes formed the basis of a coding framework which was collaboratively applied to the remaining transcripts by two team members (Y.L. and L.M.), who made adaptations to the coding framework as needed. Coding of earlier transcripts was checked and updated following adaptations to the coding framework. Generation of themes was iterative, whereby codes were reviewed and collated to form initial themes and sub-themes which were then continually checked against the dataset and refined until the team members performing the analysis were satisfied that the final set of themes and their descriptors were representative of the data. All team members reviewed drafts and the final organization of themes and sub-themes.

## RESULTS

A total of 18 stakeholders (7 project partners and 11 project implementers) across the four citizen science projects participated in an interview. Participants included policy makers (*n* = 2; project partners), senior managers (*n* = 3; project partners), health promotion officers (*n* = 2; project partners), academic researchers (*n* = 6; project implementers) and local government staff (*n* = 5; project implementers). Three main themes representing the data were developed and are outlined below.

### Theme 1: Understandings of citizen science

This theme encapsulates how participants viewed and understood citizen science and its alignment with their work. Citizen science was a new approach for most participants, with only three participants having prior experience. Project implementers overall had less familiarity with citizen science than project partners, with some first exposed to citizen science through their involvement in their respective projects. The majority of participants had experience with other public engagement approaches. Citizen science was described by both project partners and implementers as research that actively engages members of the public in issues of interest or concern to them. There was recognition that how members of the public can be involved in citizen science projects can vary. This ranged from early involvement in identifying research questions or topics of importance, to involvement in data collection, analysis and decision-making processes, and the extent to which members of the public are involved will vary depending on the purpose of the project.

A citizen science approach is one where so-called ordinary people are supported to investigate an issue in a disciplined way… So the citizen idea is linked very directly to the notion of active citizenship, not just getting citizens… to come and help with the project. The notion of citizen is the idea of the issue being of important to them and their community.—PI04

Citizen science was felt to align with participants’ personal or organizational values regarding community engagement. Some partners felt strongly about the need to collaborate with members of the public in research and practice, relating this to their personal values and/or disciplinary background. Others noted that community engagement was an organizational priority, and citizen science was viewed as a means of addressing this priority.

… my background is anthropology. So we as anthropologists fundamentally rely on what we call research informants. So you are going into any field with an understanding that the expertise sits outside; that you are not the expert. That is actually drilled into us as anthropologists: that you have to unlearn everything that you know, and you have to sit and you have to observe and you have to listen. So I really love that as a research approach; that you are recognising that expertise sits outside the academy.—PP02

So we have that strategic enabler [in relation to public engagement] within our strategic plan, it’s kind of a way citizen science provides a way to operationalise that as one of the tools that we use to really make it tangible, rather than just a sort of parenthood statement.—PP05

Although citizen science was felt to align with the work that they did, some said that they would not label their work as citizen science due to perceived similarities with other participatory approaches or how others may perceive or understand the term.

I think some people in my discipline are probably a bit put off by the science angle of it, because I think it’s [citizen science] taken up more in different sciences, not so much in the social sciences, and more in physical sciences I suppose—PI06

### Theme 2: Perceived value of citizen science in health promotion

This theme outlines factors that appealed to stakeholders about citizen science and their motivations for trialling a citizen science approach in their projects. This includes the potential for citizen science to provide access to data that would be otherwise challenging to obtain, as a tool to support more meaningful engagement with members of the community and supporting policy and practice change.

Citizen science was considered to have potential to provide a more complete understanding of preventive health issues through facilitating access to hard-to-reach data, including data that would be challenging to collect using other approaches, and through engaging multiple perspectives on health issues to inform research, policy and practice, including those who may not otherwise engage in research.

…our research design involved recruiting young people in Victoria to get them to collect screenshots of different kinds of advertising they saw in their own social media profiles. So as a research team there’s no way we could have done that, because of course every person who has a social media account, the advertising they see is tailored to them, algorithmically generated based on their interests and their ages, and different demographical characteristics. So to get what 16-year-old men in regional Victoria see, would be very different to what a 35-year-old woman in the city sees, right? So the only way to study this kind of stuff is through this kind of approach.—PI07

A consistent aim across the four projects was to improve engagement with community members to inform the development of solutions that integrate community needs and priorities. Central to how citizen science was felt to differ from other community engagement approaches was its potential to facilitate more meaningful and ongoing involvement of community members across the various stages of the research process. Participants felt that improved community engagement could generate a deeper understanding of the perspectives of community members and the issues that impact them, facilitate greater interest and support for health promotion issues and lead to members of the public advocating for improvements to their communities. Participants highlighted a need to recognize and value the skills, expertise and contributions that members of the public make to citizen science projects and ensure that citizen scientists benefit from their involvement, for example through building knowledge and awareness on the topic area and providing information and resources to support citizens to advocate for change.

So just that asking the general public to become scientists, to share in the process of research; but to actually acknowledge the kind of interest and the passions of people who don’t necessarily have formal research qualifications but are actually avid data collectors and observers in their own life because it’s what they do as a passion or a hobby.—PP02

Evaluation is really important, but I think it’s always very stakeholder and organisation driven, and obviously that is needed because we’re the ones doing the program and we’re needing to know what to do better. But I think it’s really valuable having community involved in it as much as possible. So for us in this project, it was more so around the collection of data, but even more of that; more steps in that process in regards to thinking about what questions and thinking about how we could capture it. Really empowering them, because I think the more you get community involved in any aspect of your program, whether it’s the planning or the evaluation side, I think they take a little bit more responsibility and also think about themselves and their outcomes, but also outcomes for their community, and start to get a bit more passionate at a community perspective what we could be doing to help them.—PI10

Participants discussed the goal of generating and disseminating research to support policy and practice change, including identification of priority areas for action. For this reason, it was reported that project teams actively identified strategies to bring together different stakeholders to address project findings, investigate the most appropriate way of communicating findings for impact and identify opportunities for funding to support action.

If citizens are doing this work, what can reinforce their efforts? We don’t want them just to have a lovely description … for the bigger actions, we’re in a position where we can advocate for some funding. And it is likely that we will have an infrastructure grant round. And even in thinking about this project, earlier on, I thought, we really need an infrastructure grant round, but we also need a process that prioritises action at a local level, but fits into the priorities of communities, and looks at the barriers to them being more physically active, for example. So I think this tool will be enormously helpful to support local communities to prioritise action.—PP01

Supporting and mobilizing advocacy by community members was another goal of several projects to support policy and practice change. Through engagement in projects, participants felt that citizen scientists would develop a sense of ownership over issues, and consequently, motivation to act. However, one project recognized that support for advocacy was not built into their project design.

And ultimately long term, we’d like to think that the data, the community can use it to advocate for change really. To bring about some of the changes that they identify as priorities… we haven’t in this project had the capacity to do any support for our communities, around what that advocacy might look like, or to guide that in any way, shape or form, or do any capacity building. But having said that, most of our community members were connected to other organisations from whom advocacy would have been part of what they were aiming to do in the general sense. So what this did was give them some data around this particular focus area that they could potentially use.—PI01

### Theme 3: Feasibility of applying citizen science approaches

This theme considers factors that supported or inhibited the application of citizen science in policy and practice contexts, including navigating working relationships and governance, capacity and resourcing to deliver projects, recruitment and engagement of citizen scientists, ethical considerations and data quality and quantity.

#### Working relationships, governance and resourcing of citizen science projects

Partners reported a range of considerations relating to working collaboratively with different organizations to deliver citizen science projects, with several participants acknowledging that a citizen science approach was very different to how their organizations would typically work. Navigating different, and occasionally competing, ways of working, timelines and priorities required compromise and flexibility. Additionally, local government implementers faced new research processes, such as ethics submissions, which required additional planning and support from project partners.

They had a few pressures because their funding is 12 months, so they wanted to start straight away and didn’t understand the process [of applying for ethical approval] and what we have to do. So I think some of that was challenging as well. There’s a whole lot of factors that you have to think about before starting off an approach like this that’s pretty much brand new to lots of organisations we would work with.—PP07

A few partners felt that getting buy-in from implementation partners to use a citizen science approach was challenging, and others experienced challenges in managing roles and responsibilities of collaborating organizations. Strategies described to overcome these challenges included sharing resources and information about the potential benefits of citizen science and regularly revisiting roles and responsibilities. Despite these challenges, participants reflected on the strengths afforded by working collaboratively, such as having extra resources and perspectives contributing to projects.

…the support from the policy partners – in our case I think that’s something that’s really helped, we haven’t had to convince anyone that this is a good thing to do and they can really see – it’s actually been really nice for us, because they can clearly articulate how useful this information is for them.—PI03

Most participants relayed challenges relating to resourcing and their capacity and expertise to deliver citizen science projects, acknowledging the time and skills required to apply a new approach, especially in a resource-constrained environment. Sharing insights and learnings across the four citizen science projects and capacity building support received as part of the broader Citizen Science in Prevention Project was felt to mitigate this to some extent but there was a recognized need for broader capacity building efforts to enable citizen science approaches to be embedded within their organizations more widely. A few participants reflected on their experience using citizen science as not necessarily being less resource intensive than traditional methods, but that resources were used in different ways. For example, while data collection was seen to use fewer resources in some projects, this was offset by the time spent supporting and interacting with citizen scientists or analysing large volumes of data collected.

So in a resource use, I mean, it’s interesting. Has it saved us time and money? I think would be a – I’m not sure. I mean, as a researcher, you could possibly have run around and filled in the segment audits, and taken photos, and done all of that maybe in a day. But of course, they’re not our towns, and they’re not the – so, I guess, I’m not convinced it necessarily saved on time and resources. I think it was just that they were used in a different way.—PI01

#### Recruitment and engagement

Although most participants anticipated recruitment of citizen scientists to be a challenge, the two projects that had completed recruitment reported finding it easier than anticipated to attract citizen scientists, which they attributed to their enrolment methods and recruiting through community champions. However, one reported difficulty with sustaining engagement and another in recruiting citizen scientists that were representative of the community.

Once we had them, in terms of that onboarding survey, we had between 85% to 100% retention in the various waves. It was between inviting them and them completing survey one, that was closer to 70–75% retention, and then obviously there’s a big drop off after they get the gift card, after they’ve finished the formal week of data collection and do that survey… But within the actual confines of the project, from survey one to two, with the week of data collection in the middle, we were really impressed by how many stayed with it.—PI07

Both projects that had yet to complete recruitment also viewed engaging a diverse and representative sample as a potential challenge in addition to concerns about low or insufficient participation rates and/or poor retention, particularly with the risk of disruptions from COVID-19.

The risks will be about getting the numbers and getting the commitment.—PI11

… the scope of the information that you will get, is pretty dependent on community interest in the project. You’re not going to get representative samples, because you’re going to get a snapshot of people who are generally interested in research and public health. That’s another challenge.—PI02

#### Ethical issues

Two project teams reflected on the importance of ensuring reciprocity or mutual benefit for citizen scientists who contribute to projects, widely considered a fundamental principle of citizen science, and the onus that this placed on them. For example, one participant felt failing to ‘close the loop’ or act upon findings in a timely manner may risk damaging the relationship with citizen scientists. An important strategy raised in light of these discussions was setting clear expectations at the outset of what the project is likely or able to deliver and asking citizen scientists what they would like to get out of their involvement.

I think the risk is that, if we are asking of so much time from the participants, doing a number of data collection events, then focus groups. And if they don’t, I guess, one, get the information fed back to them, I think that’s a risk, like in a timely manner. And if they’re not – and if there’s no change that comes out of some of the things that they say, I think that’s also a risk as well. So everyone needs to be – like it’s all good to say, you’re going to be involved in the whole process. But whether [implementer organisation] then make those changes at the end, or not, is out of the hands of us as a [organisation] and the community. Yeah. So that’s the biggest risk for me, that nothing comes out of it.—PP09

Related to this, remuneration of citizen scientists was considered essential by partners and implementers in one project to incentivize participation and acknowledge and value the significant contribution they were asking of their volunteers. This prompted discussions among the team to weigh up what compensation felt ‘fair and reasonable’ and would allow them to recruit a sufficient number of citizen scientists within their budget without undermining citizen’s motivation to take part.

It’s got to be intrinsically appealing as well as there be a reasonable incentive. And I see the incentive much – to me, in all of the participatory work I do, I nearly always pay my participants. I always do. I see the payment as important in terms of valuing the contributions these people make to a project. So if you’re saying like these young people know things about this world that only they know, then I feel like if I’m going to pay an RA [Research Assistant] to do something on the project then I ought to pay these people because they have expertise and skills and we should value that, particularly if it’s young participants.—PI06

Another ethical consideration raised by participants related to protecting the safety and wellbeing of citizen scientists. This included ensuring citizen science activities, such as data collection, protected the welfare and privacy of citizen scientists and did not put them or others at risk of harm. For example, one participant spoke about the challenge of determining what types of health information were appropriate to ask citizen scientists to collect from other members of their community, acknowledging that certain topics felt ‘off limits’.

So just the content of what it is you would like to use citizen science for, it feels like only fits in a very safe realm, anything to do with mental wellbeing, loneliness, any social isolation, any of those sorts of realms our community partners are very uncomfortable, because they are concerned about the safety of individuals. That’s really where they are coming from, which I understand. But yes, if we were doing something that was, I think that might be why the environmental examples of counting frog calls and working out how the frogs are going, it’s so distant from the individual collecting the data, whereas in the area of prevention I’m finding it so much more sensitive area. That would be my biggest observation, it’s more challenging.—PP05

#### Data quality, quantity and interpretation

Both projects that had completed data collection reflected on the rich quality of data they were able to yield. One project reported a larger than anticipated amount of data collected which contributed to a richer and more comprehensive understanding of the topic under investigation. Involving citizen scientists in the sensemaking of data was felt to complement and add to the data collected in another project, allowing for the generation of richer insights and priorities for action.

Because even though our citizen scientists collected data through auditing parts of their town, if we had just really looked at that quantitative data that they collected and left it at that, I think our findings would have been a bit different to what they have been, which is where we’ve gone back with that data and used it as a prompt, a discussion prompt in a workshop. And so a real benefit of it is that not only did they collect that audit data which was really helpful… But then the getting back together and using that as a way to discuss what’s going on really made sense of some of that data and I think doing one of those things alone by themselves wouldn’t have given us that richness that we have been able to generate through combining those two methods.—PI03

However, failure to capture quality data was a risk perceived by three participants, which they felt could occur with insufficient recruitment of citizen scientists or if citizen scientists provided incomplete data.

## DISCUSSION

Drawing on four citizen science projects across Australia, this study explored stakeholders’ perceptions and early experiences of adopting a citizen science approach as part of their work. Our findings highlight feasibility considerations for the broader implementation of citizen science approaches in policy and practice settings in health promotion. While many of the participants in this study felt that citizen science had potential to engage community members more meaningfully in health promotion, there was variation in stakeholders’ familiarity and understandings of how citizen science approaches could benefit their work and the areas of health promotion that may be amenable to a citizen science approach. This finding adds to wider calls for guidance to support policy and practice stakeholders in determining when to use citizen science over other community engagement methods and for what purpose, including the benefits and challenges of citizen science approaches (Pytlikzillig and Tomkins, 2011; Domecq *et al.*, 2014; Marks *et al.*, 2023). In response to these findings, our team developed a suite of resources including an introductory factsheet, videos and a case study series available on our project website aimed at policy and practice stakeholders considering using citizen science approaches in their work (The Australian Prevention Partnership Centre, 2021).

Our research found that the implementation of citizen science approaches required, for some, a shift in ways of working or models of engagement from more *ad hoc* community engagement to an approach perceived as more structured and intensive. Citizen science required similar or more resources than other approaches, and greater support for implementation, posing a challenge for wider implementation in policy and practice settings, which are often resource constrained. Within this study, collaborative relationships and capacity building support provided through the Citizen Science in Prevention Project mitigated these challenges, at least in part. These findings align with capacity building literature in citizen science (e.g. Richter *et al.*, 2018; Balestrini *et al.*, 2021), and illustrate the importance of engaging different actors and identifying resource needs to support implementation. Research indicates the importance of shared motivation and purpose in collaborative relationships (Boaz *et al.*, 2018; Laird *et al.*, 2020; Alderwick *et al.*, 2021). Newman *et al.* argue that capacity and collaborative relationships could be supported in citizen science through establishing professional associations, such as communities of practice, that reach broad stakeholders and organizations and that support the provision of infrastructure and resources (Newman *et al.*, 2012). In response to our emerging findings and the broader literature, we established a community of practice and developed guidance and practical resources to support the implementation of citizen science in disease prevention and health promotion, which can be found on our project website (The Australian Prevention Partnership Centre, 2021). Our findings also indicate a need for more targeted support to facilitate the implementation of citizen science approaches, for example through building staff capability and expertise in citizen science approaches within policy and practice organizations. As part of our program of work, we have delivered capacity building presentations, workshops and provided individual support and mentoring to stakeholder teams in the application of citizen science approaches in policy and practice settings. These capacity building activities have been evaluated and will be reported separately (Rowbotham *et al*., 2023).

Remuneration of citizen scientists was an important feasibility and sustainability consideration for some organizations. There was a concern that failure to appropriately acknowledge the contributions of citizen scientists could serve to damage the project team–citizen scientist relationship. These findings reflect a key principle of citizen science around ensuring reciprocity (Australian Citizen Science Association, 2018) and add to broader debate in the literature about compensation of citizen scientists. Some argue that financial remuneration of citizen scientists is inappropriate because it could compromise intrinsic motivation to participate. Others contend that non-payment is exploitative, and that appropriate payment values the contributions of citizen scientists, addresses barriers for participation among underserved groups, sustains engagement and improves the quality of data obtained (Resnik *et al.*, 2015; Alizadehtazi *et al.*, 2022). Smith *et al.* argue that financial compensation may not be feasible or appropriate for all projects, and that contributions of citizen scientists can be valued through other mechanisms, such as building the knowledge and capacity of citizen scientists, recognition of academic contributions through co-authorship of publications and invitations to speak at conferences (Smith *et al.*, 2019). Our findings suggest a need to build in mechanisms and resources to ensure reciprocity at the project planning stage, including establishing an understanding of what citizen scientists would like to get out of their involvement. A promising avenue of future research would be to examine whether and how remuneration of citizen scientists impacts feasibility concerns identified in this study, such as recruiting a diverse and representative sample, sustaining engagement and data quality.

### Strengths and limitations

The researchers conducting interviews were embedded evaluators rather than objective outsiders. Whilst this could be considered a limitation due to the potential risk of participants feeling more inclined to speak positively about their experiences of citizen science, we would argue that our position as embedded evaluators facilitated open dialogue between the research team and study participants and reflection and shared learning across the four projects in line with a developmental evaluation approach (Patton, 2010; Van Stolk and Ling, 2020). The resulting data represent a balanced view of the process of implementation, suggesting that participants were comfortable talking openly about their experiences. The four citizen science projects included in this evaluation varied in scope and were at different stages of development. While this limited the direct comparisons that could be made across the projects, the variation reflected the different levels of engagement in citizen science that is typical of usual practice and revealed the diverse factors that hindered or facilitated progress at different stages. Finally, while our findings provide important insights on the feasibility of citizen science approaches in policy and practice settings in health promotion, there is a need for robust evaluation of the impacts of citizen science projects in these contexts to inform their wider application. An impact evaluation is underway and will be reported separately.

## CONCLUSION

As interest in citizen science continues to grow in policy and practice settings, our findings indicate feasibility for the wider application of these approaches in disease prevention and health promotion and highlight considerations for their expanded use in these settings. To support broader acceptance and use of citizen science approaches in health promotion, tools and resources to build capacity in policy and practice contexts are needed, as well as a comprehensive understanding of the impacts and costs of citizen science approaches.

## References

[CIT0001] Alderwick, H., Hutchings, A., Briggs, A. and Mays, N. (2021) The impacts of collaboration between local health care and non-health care organizations and factors shaping how they work: a systematic review of reviews. BMC Public Health, 21, 753. 33874927 10.1186/s12889-021-10630-1 PMC8054696

[CIT0002] Alizadehtazi, B., Woerdeman, S., Tangtrakul, K., Gussenhoven, A., Mostafavi, N. and Montalto, F. (2022) Recruiting, paying, and evaluating the experiences of civic scientists studying urban park usage during the beginning of the COVID-19 Pandemic. Frontiers in Sustainable Cities, 109.

[CIT0003] Australian Citizen Science Association. (2018) 10 Principles of Citizen Science. Australian Citizen Science Association, Australia.

[CIT0004] Australian Institute of Health and Welfare. (2020) Australia’s Health 2020 Snapshots. Australia’s Health Series No. 17. AIHW, Canberra.

[CIT0005] Balestrini, M., Kotsev, A., Ponti, M. and Schade, S. (2021) Collaboration matters: capacity building, up-scaling, spreading, and sustainability in citizen-generated data projects. Humanities and Social Sciences Communications, 8, 169.

[CIT0006] Boaz, A., Hanney, S., Borst, R., O’shea, A. and Kok, M. (2018) How to engage stakeholders in research: design principles to support improvement. Health Research Policy and Systems, 16, 60. 29996848 10.1186/s12961-018-0337-6 PMC6042393

[CIT0007] Bonney, R., Ballard, H., Jordan, R., Mccallie, E., Phillips, T., Shirk, J. et al. (2009) Public Participation in Scientific Research: Defining the Field and Assessing Its Potential for Informal Science Education. A CAISE Inquiry Group Report. Online submission.

[CIT0008] Bonney, R., Phillips, T. B., Ballard, H. L. and Enck, J. W. (2016) Can citizen science enhance public understanding of science? Public Understanding of Science, 25, 2–16. 26445860 10.1177/0963662515607406

[CIT0009] Braun, V. and Clarke, V. (2021) Thematic Analysis: A Practical Guide. Sage, London, UK.

[CIT0010] Crosland, P., Ananthapavan, J., Davison, J., Lambert, M. and Carter, R. (2019) The health burden of preventable disease in Australia: a systematic review. Australian and New Zealand Journal of Public Health, 43, 163–170. 30830711 10.1111/1753-6405.12882

[CIT0011] De Sherbinin, A., Bowser, A., Chuang, T.-R., Cooper, C., Danielsen, F., Edmunds, R. et al. (2021) The critical importance of citizen science data. Frontiers in Climate, 3.

[CIT0012] Den Broeder, L., Lemmens, L., Uysal, S., Kauw, K., Weekenborg, J., Schönenberger, M. et al. (2017) Public health citizen science; perceived impacts on citizen scientists: a case study in a low-income neighbourhood in the Netherlands. Citizen Science: Theory and Practice, 2, 1–17. 10.5334/cstp.89

[CIT0013] Domecq, J. P., Prutsky, G., Elraiyah, T., Wang, Z., Nabhan, M., Shippee, N. et al. (2014) Patient engagement in research: a systematic review. BMC Health Services Research, 14, 89. 24568690 10.1186/1472-6963-14-89 PMC3938901

[CIT0014] Follett, R. and Strezov, V. (2015) An analysis of citizen science based research: usage and publication patterns. PLoS One, 10, e0143687. 26600041 10.1371/journal.pone.0143687 PMC4658079

[CIT0015] Frieden, T. R. (2014) Six components necessary for effective public health program implementation. American Journal of Public Health, 104, 17–22. 24228653 10.2105/AJPH.2013.301608 PMC3910052

[CIT0016] Kullenberg, C. and Kasperowski, D. (2016) What is citizen science? A scientometric meta-analysis. PLoS One, 11, e0147152. 26766577 10.1371/journal.pone.0147152 PMC4713078

[CIT0017] Laird, Y., Manner, J., Baldwin, L., Hunter, R., Mcateer, J., Rodgers, S. et al. (2020) Stakeholders’ experiences of the public health research process: time to change the system? Health Research Policy and Systems, 18, 83. 32682426 10.1186/s12961-020-00599-5 PMC7368787

[CIT0018] Marks, L., Laird, Y., Trevena, H., Smith, B. J. and Rowbotham, S. (2022) A scoping review of citizen science approaches in chronic disease prevention. Frontiers in Public Health, 10. 10.3389/fpubh.2022.743348 PMC9125037 35615030

[CIT0019] Marks, L., Smith, B. J., Mitchell, J., Laird, Y. and Rowbotham, S. (2023) The case for citizen science in public health policy and practice: a mixed methods study of policymaker and practitioner perspectives and experiences. Health Research Policy and Systems, 21, 31. 37127620 10.1186/s12961-023-00978-8 PMC10152701

[CIT0020] National Health and Medical Research Council. (2016) Statement on Consumer and Community Involvement in Health and Medical Research. National Health and Medical Research Council, Australia.

[CIT0021] Newman, G., Wiggins, A., Crall, A., Graham, E., Newman, S. and Crowston, K. (2012) The future of citizen science: emerging technologies and shifting paradigms. Frontiers in Ecology and the Environment, 10, 298–304.

[CIT0022] Patton, M. Q. (2010) Developmental Evaluation: Applying Complexity Concepts to Enhance Innovation and Use. Guilford Press, New York.

[CIT0023] Pytlikzillig, L. M. and Tomkins, A. J. (2011) Public engagement for informing science and technology policy: what do we know, what do we need to know, and how will we get there? Review of Policy Research, 28, 197–217.

[CIT0024] Qsr International Pty Ltd. (2018) *NVivo (Version 12)*.Lumivero.www.lumivero.com

[CIT0025] Resnik, D. B., Elliott, K. C. and Miller, A. K. (2015) A framework for addressing ethical issues in citizen science. Environmental Science & Policy, 54, 475–481.

[CIT0026] Richter, A., Dörler, D., Hecker, S., Heigl, F., Pettibone, L., Serrano Sanz, F. et al. (2018) Capacity building in citizen science. In Hecker, S., Haklay, M., Bowser, A., Makuch, Z., Vogel, J. and Bonn, A. (eds.), Citizen Science - Innovation in Open Science, Society and Policy. UCL Press, London, pp. 269–283.

[CIT0027] Rowbotham, S., Laird, Y., Marks, L., Walker, P., Pontifex, K., Sobhan, A. et al. (2022) Building capacity to apply citizen science approaches in policy and practice for public health: protocol for a developmental evaluation of four stakeholder-led projects. Citizen Science: Theory and Practice, 7.

[CIT0035] Rowbotham, S., Walker, P., Marks, L. Irving, M., Smith, B.J. and Laird, Y. (2023) Building capacity for citizen science in health promotion: a collaborative knowledge mobilisation approach. Research Involvement and Engagement 9, 36. 10.1186/s40900-023-00451-4 37254184 PMC10228445

[CIT0028] Shanley, L. A., Hulbert, J., Auerbach, J. and Haklay, M. (2019) CitSciDefinitions, v1.2. https://zenodo.org/badge/latestdoi/189093030

[CIT0029] Smith, E., Bélisle-Pipon, J.-C. and Resnik, D. (2019) Patients as research partners; how to value their perceptions, contribution and labor? Citizen Science: Theory and Practice, 4, 1–13. 10.5334/cstp.184 PMC7021275 32064121

[CIT0030] The Australian Prevention Partnership Centre. (2021) Harnessing the power of citizen science for prevention [Online]. https://preventioncentre.org.au/research-projects/harnessing-the-power-of-citizen-science-for-prevention/ (last accessed 3 October 2022).

[CIT0031] Tong, A., Sainsbury, P. and Craig, J. (2007) Consolidated criteria for reporting qualitative research (COREQ): a 32-item checklist for interviews and focus groups. International Journal for Quality in Health Care, 19, 349–357. 17872937 10.1093/intqhc/mzm042

[CIT0032] Van Stolk, C. and Ling, T. (2020) Understanding the practice of embedded evaluation: opportunities and challenges. In Barrados, M. and Lonsdale, J. (eds.), Crossover of Audit and Evaluation Practices: Challenges and Opportunities (1st ed.). Routledge. 10.4324/9781003021025

[CIT0033] World Health Organization. (2017) Engagement and Participation for Health Equity. World Health Organization Regional Office for Europe, Denmark.

[CIT0034] Wutzke, S., Morrice, E., Benton, M. and Wilson, A. (2017) What will it take to improve prevention of chronic diseases in Australia? A case study of two national approaches. Australian Health Review, 41, 176–181. 27305656 10.1071/AH16002

